# Supplementary material for: Numerous inequalities and related communications accompanying discrete divergence models in probability spaces
Source: PLoS One. 2026 Feb 13;21(2):e0341742. doi: 10.1371/journal.pone.0341742 (PMC12904446; doi:10.1371/journal.pone.0341742)
Supplement: Attachment — Submitted filename: Responses to Reviewers.docx [file pone.0341742.s002.docx]

**Reviewer #1, Concern #1: Language and Style**

The reviewer suggested that the abstract and introduction include dense and complex sentences, recommending grammatical refinement and simpler phrasing. The example given was to replace “the analysis of inequalities allows for the recording of ideal coding schemes” with “the analysis of inequalities aids in the formulation of optimal coding schemes.”

**Author response:**

The reviewer is thanked for this helpful observation. We agree that the simplicity of some of the phrasing will increase clarity and readability, particularly for those from more applied backgrounds.

**Author action:**

Grammar has been corrected and, in some cases, the sentence structure has been simplified in the abstract and introduction. Phrases that were too complicated were avoided and simplified to make it easier to flow and to understand. For example, from the statement in the review about this sentence, we rephrased it as "analysis of inequalities helps with stating the best coding schemes." Exact modifications were made across the manuscript to improve precision of the language and readability and were highlighted with red colour.

**Reviewer #1, Concern #2: Notation and Formatting**

The reviewer mentioned that several mathematical expressions could benefit from clearer formatting and that consistent notation for divergence functions and entropy should be ensured throughout the manuscript.

**Author response:**

We appreciate the reviewer’s attention to detail. We agree that uniform notation and formatting are important for mathematical rigor and reader comprehension.

**Author action:**

All mathematical symbols, equations, and divergence functions have been reviewed and reformatted for consistency. The notation for divergence measures, entropy functions, and probability distributions has been standardized throughout the paper. Special care was taken to ensure that symbols such as (D(P;Q)), (I(P;Q)), and (H(P)) are consistently used, and that equation numbering and alignment follow a uniform style.

**Reviewer #1, Concern #3: Contextual Examples**

The reviewer suggested including one or two applied examples (e.g., image fusion, data classification, or sensor networks) to demonstrate the real-world relevance of the proposed inequalities and divergence models.

**Author response:**

We appreciate this suggestion made by the reviewer. We strongly agree that linking theoretical results to the practical effects would make the paper more interdisciplinary relevant.

**Author action:**

Practical applications are discussed in subsection 2.8, which has been added in the discussion section. To show where the inequalities and divergence models can be utilized in practice we have introduced a few succinct contextual examples from image fusion and highlighted them using red colour.

**Reviewer #1, Concern #4: Section Titles and Flow**

The reviewer recommended dividing longer sections into more focused subsections (for example, “Applications to Shannon Entropy,” “Generalized Divergence Functions,” “Symmetric Divergence Measures”) to enhance readability and organization.

**Author response:**

We appreciate the reviewer for this helpful comment. Yes, the MS does have long subsections that bring in different theories under one heading. We think the paper would have been more readable if it were subdivided into subsections with clear headings, since this would have helped guide the reader through a logical move from general concepts to specific results.

**Author action:**

Abstract: stations have been reviewed and suitable subsection titles proposed for more accurately reflecting the content. This structure has been improved for a logical order — from basics, to inequalities, to applications of divergence measures. Suggested subsection headings for clarity and ease of reference with red colour for changes highlighted

**Reviewer #1, Concern #5: Overall Readability and Accessibility**

The reviewer’s general comments emphasize improving clarity and accessibility while maintaining mathematical rigor.

**Author response:**

We appreciate the positives in the review and the constructive comments made. We are aware that a balance between mathematical depth and clarity is needed to appeal to a wide audience.

**Author action:**

The entire manuscript also received its final review for proofreading and formatting in addition to the specific revisions shown above. We deleted any repetitive phrases, improved transitions between sections, and otherwise toned the language to be professional but accessible.

**Reviewer #2, Concern #1: Clarity and Practical Intuition**

**Reviewer comment:**

While the manuscript offers a comprehensive mathematical treatment of inequalities related to discrete divergence models, the scope is very theoretical and dense. The introduction and discussion could improve clarity by adding more intuitive explanations and examples of practical applications to help readers from applied fields grasp the significance.

**Author response:**

We appreciate this feedback from the reviewer. This style of commenting gives visceral context to mathematical rigor that we believe is necessary for reaching a wider audience, including those in applied disciplines.

**Author action:**

The introduction has been modified to provide more clarity on the motivation of divergence models and their relevance in real-world applications in communication theory and data analysis. We have also included a detailed numerical example (Example 2.8, “Application to Image Fusion”) to show the application of the derived inequalities when assessing information preservation in the image fusion problem. It illustrates a simple example wherein the proposed theoretical framework has some practical utility.

**Reviewer #2, Concern #2: Novel Contributions and Differentiation from Prior Work**

**Reviewer comment:**

The work builds upon known divergence measures such as Kullback–Leibler and Rényi divergences. However, the manuscript would benefit from more explicit highlighting of its novel contributions, distinguishing its new inequalities or generalizations clearly from prior art.

**Author response:**

Thanks to this very useful suggestion by the reviewer. We can only agree with the reviewer that it is important to concisely and clearly make this distinction in the introduction, allowing readers to understand the novelty of the theoretical results with respect to previously proposed models.

**Author action:**

Introduction Section has been concluded by adding a new paragraph called Research gap and motivation. The highlighted with green colour this section clearly points out the shortcomings in the previous study in a way that most of the divergence measures are discussed in isolation from one another without global inequalities or some generalized interrelations between these measures.

**Reviewer #2, Concern #3: Dense Theoretical Sections and Lack of Structural Separation**

**Reviewer comment:**

Some sections are overly technical, with long dense formulas and theoretical proofs. The manuscript would gain from better structuring, including summarizing key results in tables or diagrams, and separating theoretical developments from applied implications more distinctly.

**Author response:**

We thank the reviewer for this constructive feedback. We agree that improved structure and visual organization make dense theoretical material more accessible.

**Author action:**

Following both reviewers’ suggestions, the main body of the paper has now been divided into clear subsections (e.g., Applications to Shannon Entropy, Symmetric Divergence Measures and Numerical Example).

**Reviewer #2, Concern #4: Referencing and Integration with Literature**

**Reviewer comment:**

The referencing is extensive but could be more critically integrated. It is suggested to better contextualize the new inequalities within existing frameworks and to discuss more recent related works, if any, to position the study in the current research landscape.

**Author response:**

We appreciate the reviewer’s recommendation. Since embracing a more critical approach to how we use references improves the story telling and places our work in context with others, we acknowledge this.

**Author action:**

Incorporated recent works on entropy-type divergence models and inequalities into the revised Introduction and Discussion. Each cited study is now provided communication context regarding how it supports or contrasts with our proposed inequities. For more detail and background, see Shannon [24], Rényi [23] and recent generalizations [e.g., Kumari & Sharma [13], Torra et al. Note that the methods have been refined from what initially was described [30] to illustrate the manner in which the present results developed.

**Reviewer #2, Concern #5: Lack of Experimental or Numerical Validation**

Reviewer comment:

The paper is heavily theoretical without experimental validation, simulations, or examples showing the practical utility of the new inequalities in real-world scenarios. Including such elements would substantially strengthen the impact.

**Author response:**

We agree that providing a demonstrated practical relevance provides a greater influence of theoretical work.

**Author action:**

To illustrate the usage of the proposed inequalities in image fusion, an illustrative numerical example (Example 2.8) is given. This case calculates Kullback–Leibler, Rényi, and J-divergences of discrete probability distributions obtained from sample image data and demonstrates the behavior of inequalities in the practice. By adding this, I directly respond to the reviewer's concern that the framework is too abstract while simultaneously demonstrating that it leads to measurable outcomes.

**Reviewer #2, Concern #6: Language and Readability**

**Reviewer comment:**

Although mathematically rigorous, some parts of the manuscript suffer from verbose and complicated language, which could be simplified for readability without losing precision.

**Author response:**

We appreciate the reviewer’s observation. We admit that short and simple writing is one of the keys to good presentation.

**Author action:**

The manuscript has been redraft and perused painstakingly. Long and convoluted sentences were simplified, redundancy was eradicated, and the flow of ideas was enabled. This is to make sure that the document is readable without losing any technical inherent precision.
