# Supplementary material for: Numerous inequalities and related communications accompanying discrete divergence models in probability spaces
Source: PLoS One. 2026 Feb 13;21(2):e0341742. doi: 10.1371/journal.pone.0341742 (PMC12904446; doi:10.1371/journal.pone.0341742)
Supplement: Attachment — Submitted filename: Response to Reviewers.docx [file pone.0341742.s003.docx]

**Reviewer #2, Concern #1: Language and Typos**

The reviewer notes that the overall language of the manuscript has improved and is now clear. However, a few minor issues remain,

Language and Typos:

The language of the paper sounds good now. However, I have noticed two typos:
• The first line from the paragraph after (1.5): “different instigators …”. I believe authors mean investigators.

• The last line from the last paragraph after (1.12): “To satisfy our aim, we need the following standard findings from Ash[1] and Cover and Thomas [2]:”. The reader expects to read about the findings on the next page, however; a new section is presented on that page.
• “Result 2.9: If a and b be positive...”. Correct “be” to “are”.

**Author response:**

The author expresses gratitude to the reviewer for positively assessing the updated text and identifying lingering typographical and structural concerns. We agree that addressing such subtleties.

**Author action:**

All of the points raised by the reviewer have been addressed in the revised article. The term "instigators" has been substituted with "investigators" in the paragraph that follows equation (1.5). The statement relating to the standard results from Ash [1] and Cover and Thomas [2] has been moved to come directly before the presentation of those findings, following the "Research Gap and Motivation" section, to better connect reader expectations with the forthcoming material. Furthermore, the grammatical mistake in Result 2.9 has been fixed by changing "be" to "are." These edits enhance the manuscript's readability, consistency, and presentation quality.

.
